# Leukocyte-Dependent Regulation of Cardiac Fibrosis

**DOI:** 10.3389/fphys.2020.00301

**Published:** 2020-04-08

**Authors:** Ama Dedo Okyere, Douglas G. Tilley

**Affiliations:** Center for Translational Medicine, Lewis Katz School of Medicine, Temple University, Philadelphia, PA, United States

**Keywords:** cardiac fibrosis, neutrophil, monocyte macrophage, mast cells, lymphocytes, dendritic cells, eosinophils, inflammation

## Abstract

Cardiac fibrosis begins as an intrinsic response to injury or ageing that functions to preserve the tissue from further damage. Fibrosis results from activated cardiac myofibroblasts, which secrete extracellular matrix (ECM) proteins in an effort to replace damaged tissue; however, excessive ECM deposition leads to pathological fibrotic remodeling. At this extent, fibrosis gravely disturbs myocardial compliance, and ultimately leads to adverse outcomes like heart failure with heightened mortality. As such, understanding the complexity behind fibrotic remodeling has been a focal point of cardiac research in recent years. Resident cardiac fibroblasts and activated myofibroblasts have been proven integral to the fibrotic response; however, several findings point to additional cell types that may contribute to the development of pathological fibrosis. For one, leukocytes expand in number after injury and exhibit high plasticity, thus their distinct role(s) in cardiac fibrosis is an ongoing and controversial field of study. This review summarizes current findings, focusing on both direct and indirect leukocyte-mediated mechanisms of fibrosis, which may provide novel targeted strategies against fibrotic remodeling.

## Cardiac Fibrosis

The healthy heart is supported by a distinct network of extracellular matrix (ECM) proteins, which function in part to preserve chamber structure and aid in cardiac cell communication (Lu et al., 2011; Frangogiannis, 2017). Fibroblasts resident to the heart are primarily responsible for maintaining the ECM, which typically consists of roughly 80 percent type I collagens, 10 percent type III collagens, and a combination of proteoglycans, glycoproteins, and glycosaminoglycans (Kong et al., 2014). The cardiac ECM is highly dynamic, and it’s homeostasis is preserved through a few mechanisms: a strict balance between protein synthesis and degradation, as well as protein reorganization within the network (Lu et al., 2011). However, virtually any injury to the heart will activate fibrosis, or a dysregulated and enhanced growth of the ECM milieu, since the adult heart holds limited capacity for regeneration (Humeres and Frangogiannis, 2019). For instance, following a myocardial infarction (MI), the severe loss of cardiomyocytes triggers increased matrix production in an effort to replace lost and damaged tissue (Frangogiannis, 2019a). Further, other pathologies like pressure overload due to hypertension, volume overload, diabetes, obesity, and even aging can stress any chamber of the heart, therefore leading to fibrotic growth around the vasculature and within the interstitium (Aguiar et al., 2019; Frangogiannis, 2019a). Although this matrix growth initially serves as a form of repair, it can quickly become detrimental (Frangogiannis, 2019a). An exaggerated production of collagens can result in myocardial stiffening, which disrupts cardiac compliance and ultimately decreases systolic and diastolic function (Kong et al., 2014; Tallquist and Molkentin, 2017). Importantly, it has recently been shown that severe cardiac fibrosis, especially when accompanied by heart failure (HF) is associated with increased mortality in patients (Aoki et al., 2011; Gyongyosi et al., 2017). While there are currently treatment options that help in managing the symptoms of heart disease, there are no effective therapies targeted at controlling pathological fibrosis. Therefore, an in-depth discussion, focusing on the mechanisms that contribute to the genesis of fibrosis is extremely necessary (Gyongyosi et al., 2017; Tallquist and Molkentin, 2017; Aghajanian et al., 2019; Humeres and Frangogiannis, 2019).

In past years, numerous rigorous studies have identified the resident cardiac fibroblast as a crucial cell effector of pathological fibrosis (Davis and Molkentin, 2014; Kanisicak et al., 2016; Tallquist and Molkentin, 2017; Fu et al., 2018; Ivey et al., 2018). Due to injury, resident fibroblasts proliferate, and differentiate into mature cardiac myofibroblasts, which, through *de novo* expression of α smooth muscle actin (αSMA) can contract to support wound healing (Davis and Molkentin, 2014). Activated myofibroblasts also contribute to fibrosis by secreting copious amounts of matrix protein, which then overwhelms the ECM (Davis and Molkentin, 2014; Stempien-Otero et al., 2016; Tallquist and Molkentin, 2017). Due to the hearts cellular complexity, additional sources, including vascular and bone marrow derived cells have been considered for their contribution to myofibroblast differentiation and fibrosis (Kanisicak et al., 2016; Travers et al., 2016; Tallquist and Molkentin, 2017; Fu et al., 2018). This review will focus on the leukocyte dependent regulation of fibrosis since it has been well reported that the activation of resident fibroblasts into myofibroblasts is heavily influenced by changes in the myocardial environment (Davis and Molkentin, 2014; Stempien-Otero et al., 2016). This is important to note, as injury results in damaged cardiomyocytes which incite inflammation and promote a large expansion and infiltration of leukocytes (Swirski and Nahrendorf, 2013; Grisanti et al., 2016a, b; Swirski and Nahrendorf, 2018). These infiltrating leukocytes occupy spaces near sites of injury, and function through cytokines and growth factors secretions, which contribute to changes in myocardial environment (Swirski and Nahrendorf, 2013, 2018). As such, the role of leukocytes in cardiac fibrosis has been a longstanding research focus. Since leukocytes drastically expand in number following injury, and exhibit high cellular plasticity, earlier studies sought to determine if, and to what extent, leukocytes serve as additional cell sources for myofibroblast transdifferentiation (van Amerongen et al., 2008; Alex and Frangogiannis, 2018). More recent studies have focused on both the direct and indirect roles of distinct leukocyte subsets in cardiac fibrosis. Below, we will examine the current understanding of leukocytes in cardiac biology, focusing on how they may regulate cardiac fibrosis, and examining if they can be targeted to control fibrosis.

## Leukocytes in Cardiac Physiology and Pathology

Any tissue injury leads to inflammation, which engages a collective of white blood cells termed leukocytes (Geissmann et al., 2010; Kondo, 2010). The primary function of leukocytes is to sense and respond to pathogens or damage in order to suppress any additional danger (Kondo, 2010). Leukocytes exist in various subsets, and can be classified as being of either lymphoid or myeloid origin (Kondo, 2010). Their origin and subsequent gene expression profiles are key to their function (Geissmann et al., 2010; Kondo, 2010). All leukocytes initially derive from hematopoietic stem cells, which differentiate into either lymphoid or myeloid progenitors (Kondo, 2010). Lymphoid progenitors give rise to B and T lymphocytes, natural killer cells, and some subsets of dendritic cells (Kondo, 2010). Myeloid progenitors are precursors for megakaryocytes, erythrocytes, granulocytes, and monocytes (Kondo, 2010). Seminal studies in recent years have provided ample evidence for the roles and origins of leukocytes both in the steady-state and injured heart (Epelman et al., 2014; Swirski and Nahrendorf, 2018).

Through flow cytometry and single cell RNA sequencing technologies, researchers have established the presence of many leukocyte subsets in the injured and healthy heart, with most of them being macrophages (Epelman et al., 2014; Swirski and Nahrendorf, 2018; Aguiar et al., 2019; Dick et al., 2019). Further studies using adult mouse hearts have allowed us to gain greater appreciation for the existence and diversity of these cardiac macrophage populations (Swirski and Nahrendorf, 2018). A minority of cardiac macrophages, expressing chemokine receptor 2 (CCR2), stem from circulating monocyte precursors (Epelman et al., 2014; Bajpai et al., 2018). However, additional cardiac macrophage subsets have been shown to inhabit the heart early, and persist throughout adulthood via proliferation (Epelman et al., 2014; Bajpai et al., 2018; Dick et al., 2019). Functionally, cardiac macrophages are thought to act as pathogen sensors, but they may also take more active part in myocardial electrical conduction (Ma et al., 2018; Swirski and Nahrendorf, 2018). Interestingly, recent findings suggest an abundance of connexin 43-expressing macrophages in the atrioventricular (AV) node, which exhibit an ability to couple to cardiomyocytes and alter their membrane potential (Hulsmans et al., 2017). Other leukocyte populations in the steady-state heart include lymphocytes, dendritic and mast cells (Choi et al., 2009; Swirski and Nahrendorf, 2018). The complete function of these cells is still not entirely understood, however, mast cells have been shown to be stores of preformed cytokines, which may prove beneficial at the onset of injury (Frangogiannis et al., 1998).

Cardiac fibrosis is a typical feature of ageing and injury, both of which are known to trigger leukocyte expansion and inflammation within the heart (Epelman et al., 2015; Lu et al., 2017; Suetomi et al., 2018; Aguiar et al., 2019). For instance, a plethora of studies have revealed the rapid and continuous innate immune response that follows acute ischemic injuries. Neutrophils and inflammatory monocytes/macrophages infiltrate the myocardium in response to endogenous damage-associated molecular patterns (DAMPs) where they can effectively clear dying cells and regulate inflammation through cytokines, chemokines, and reactive oxygen species (Forte et al., 2018). Following this pro-inflammatory phase, additional monocyte subsets (Ly6C^*lo*^) are recruited to promote transition to injury repair through secretion of pro-repair factors (Nahrendorf et al., 2007; Forte et al., 2018). Cluster of differentiation (CD) 11b^+^/CD11c^+^ dendritic cells have also been shown to accumulate in the border zone post-myocardial infarction (MI), peaking around 7 days post-injury (Gallego-Colon et al., 2015). Dendritic cells are thought to aid in preserving left ventricle (LV) function activate T lymphocyte subsets through antigen presenting functions (Forte et al., 2018). The role of adaptive immunity in response to ischemic injury is still being uncovered, however, recent studies suggest the involvement of both B and T lymphocytes in cardiac remodeling post-MI (Forte et al., 2018). For instance, CD4^+^ T cells have been shown to invade the heart by 7 days post-MI, where they are presumed to modulate LV function (Hofmann et al., 2012). Similarly, B cell volumes expand following MI, reaching a peak around 5–7 days post-injury (Yan et al., 2013; Zouggari et al., 2013). Functionally, B cells may contribute to the regulation of inflammatory gene expression post-ischemic injury (Zouggari et al., 2013). The innate and adaptive immune response have also been characterized in ageing and injuries of non-ischemic etiology. For instance, mouse models of advanced ageing, and hypertension through high salt, unilateral nephrectomy, and aldosterone infusion, reveal increased neutrophil and macrophage populations within the heart (Hulsmans et al., 2018). The presence of these immune cells is associated with enhanced diastolic dysfunction. Further, pressure overload induced pathology due to transverse aortic constriction (TAC) has also been shown to result in an increased infiltration of leukocytes, both CCR2^+^ macrophages and CD4^+^ T cells (Laroumanie et al., 2014; Patel et al., 2018). The rise in leukocytes to the heart following these pathophysiological changes has prompted many researchers to question the link between inflammation and fibrosis.

## Leukocyte Involvement in Cardiac Fibrosis

**FIGURE 1:**
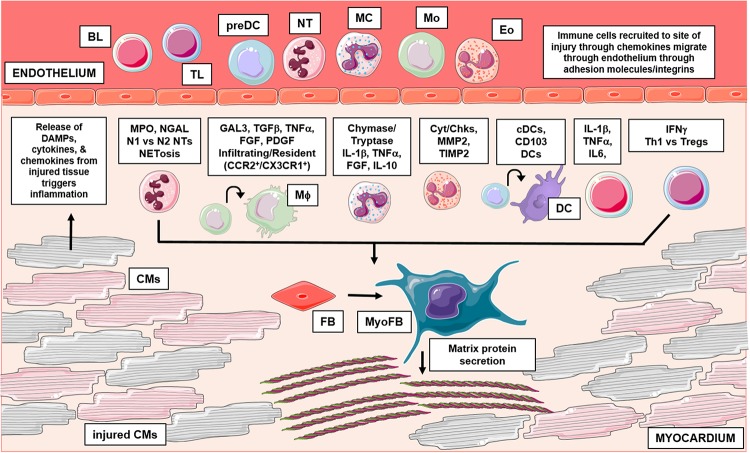
This graphical abstract summarizes the role of leukocytes in cardiac fibrosis. Following injury, damaged cardiac tissue (inclusive of all resident cell types) can release inflammatory mediators like DAMPs, which trigger an influx of leukocytes to the site of injury. Activated leukocytes then function through numerous mechanisms to regulate pathological cardiac fibrosis. Briefly, granulocytes (mast cells, eosinophils, neutrophils) have been shown to influence fibrosis in part, through their ability to secrete fibrotic mediators, regulate expression of MMPs/TIMPs and form inflammatory extracellular traps, respectively. Additionally, recent studies have identified varying subsets of macrophages and dendritic cells which differentially regulate fibrosis outcomes; cells of monocytic origin have also been examined for their direct contribution in myofibroblast differentiation. Lastly, numerous findings also implicate lymphocytes in cardiac fibrosis, these adaptive immune cells have been shown to influence remodeling outcomes through many mechanisms. BL, b lymphocyte; TL, t lymphocyte; preDC, precursor dendritic cell; NT, neutrophil; MC, mast cell; Mo, monocyte; Eo, eosinophil; Mϕ, macrophage; DC, dendritic cell; CM, cardiomyocyte; FB, fibroblast; MyoFB, myofibroblast; DAMP, damage associated molecular pattern; MPO, myeloperoxidase; NGAL, neutrophil gelatinase-associated lipocalin; N1, inflammatory NT; N2, antiinflammatory NT; NETosis, NT extracellular trap formation; GAL3, galectin-3; TGFβ, transforming growth factor beta; TNFα, tumor necrosis factor alpha; FGF, fibroblast growth factor; PDGF, platelet derived growth factor; CCR2, C-C chemokine receptor 2; CX3CR1, CX3C chemokine receptor 1; IL-1β, interleukin 1 beta; IL-10, interleukin 10; Cyt, cytokines; Chk, chemokines; MMP2, matrix metallopeptidase 2; TIMP 2, tissue inhibitor of metalloproteinases 2; cDC, conventional DC; CD, cluster of differentiation; IL-6, interleukin 6; IFNγ, interferon gamma; Th1, t helper type 1; Treg, regulatory t cells. Graphics were created using Servier Medical Art templates, which are licensed under a Creative Commons Attribution 3.0 Uniported License; https://smart.servier.com.

Fibrosis and inflammation are essential physiological processes that follow essentially all cardiac pathologies (Swirski and Nahrendorf, 2018). During inflammation, leukocytes function to resolve injury and defend the host through several eloquent mechanisms (Kondo, 2010; Bajpai and Tilley, 2018). The existing literature suggests several connections between leukocyte driven inflammation and cardiac fibrosis. Below, we consider the evidence supporting leukocyte dependent regulation of fibrosis with the ultimate goal of highlighting novel targets in the quest for combating detrimental fibrotic remodeling (Table 1 and Figure 1).

**TABLE 1:** This table lists evidence in support of how each leukocyte class may regulate cardiac fibrosis in distinct pathological contexts.

**Leukocyte class**	**Pathological context**	**Contribution to fibrosis**
Neutrophils	Ischemia	5 day post MI, increase in **CD206** and or **Arg-1** expressing neutrophils is associated with decreased myofibroblast transdifferentiation (Kain et al., 2018) 7 days post MI, neutrophils express **Fibronectin**, **Gal-3**, **Fibrinogen** which contributes to ECM reorganization (Daseke et al., 2019) 21 days post MI, loss of **MPO** reduces fibrosis (Mollenhauer et al., 2017) Post MI, loss of **NGAL** expressing neutrophils may affect dead myocyte phagocytosis and contribute to fibrosis (Horckmans et al., 2017)
	Myocarditis/DCM; Ageing	In chronic myocarditis and ageing decreased neutrophil infiltration and **NETosis**/**NET** formation is associated with reduced fibrosis (Martinod et al., 2017; Weckbach et al., 2019)
Monocytes/Macrophages	Ischemia	1–6 weeks post MI, macrophages may transition to fibroblast-like cells, capable of secreting collagen (Haider et al., 2019) **IL-10** and **TGFβ** secreting macrophages promote myofibroblasts transdifferentiation (O’Rourke et al., 2019) 1 week post ischemia/reperfusion, loss of **MCP-1** is associated with decreased macrophage infiltration and interstitial fibrosis (Frangogiannis et al., 2007) 4 weeks post MI, **Gata6** expressing pericardial macrophages limit cardiac fibrosis (Deniset et al., 2019)
	Pressure Overload	Macrophage associated **Gal-3** correlates with increased cardiac fibrosis in hypertensive rats (de Boer et al., 2009) 8 weeks following uninephrectomy and salty drinking water, loss of macrophage expressed **mineralocorticoid receptor** reduces cardiac collagen content (Rickard et al., 2009)
Mast Cells	Pressure Overload	Mast cells contribute to **PDGF-A** expression and fibrosis in the heart following TAC (Liao et al., 2010)
	DCM	Mast cells are a large source of **FGF-2**, and found within fibrotic sections of cardiac tissue (Bradding and Pejler, 2018)
Eosinophils	Myocarditis/DCM	Eosinophil depletion post myocarditis is associated with decreased levels of **MMP-2** and **TIMP-2** (Diny et al., 2017)
Dendritic Cells	Ischemia	Ablation of dendritic cells results in increased **MMP-9** and **MMP-2** activity 3–28 days post MI (Anzai et al., 2012)
	Pressure Overload	Dendritic cell ablation following TAC is associated with decreased **IL-1β** and **TGFβ** and less fibrosis (Wang et al., 2017)
	Myocarditis/DCM	**BATF3** dependent dendritic cells limit cardiac fibrosis following viral infection (Clemente-Casares et al., 2017)
B and T Cells	Ischemia	Depletion of **TNFα** T regulatory cells improves fibrosis post MI (Bansal et al., 2019)
	Pressure Overload	B cell depletion results in decreased fibrosis following TAC (Ma et al., 2019) **IFNγ** T cells promote myofibroblast trans differentiation and cardiac fibrosis (Nevers et al., 2017) CD73 expressing T cells attenuate fibrosis following TAC (Quast et al., 2017)
	Myocarditis/DCM	**TNFα** secreting B cells correlate with greater fibrosis in DCM patients (Yu et al., 2013)

*MI, myocardial infarction; CD, cluster of differentiation; Arg-1, arginase 1; GAL3, galectin-3; MPO, myeloperoxidase; NGAL, neutrophil gelatinase-associated lipocalin; NETosis, neutrophil extracellular trap formation; IL-10, interleukin 10; TGFβ, transforming growth factor beta; MCP-1, monocyte chemoattractant protein 1; Gata6, GATA binding protein 6; PDGF, platelet derived growth factor; FGF, fibroblast growth factor; MMP, matrix metallopeptidase; TIMP, tissue inhibitor of metalloproteinases; IL-1β, interleukin 1 beta; TGFβ, transforming growth factor beta; BATF3, basic leucine zipper transcription factor ATF-like 3; IFNγ, interferon gamma; TNFα, tumor necrosis factor alpha.*

### Neutrophils

Neutrophils are polymorphous nuclear leukocytes that are often recognized as the first responders to injury (Amulic et al., 2012). Neutrophils mature in the bone marrow, and their release is strictly regulated by shifting chemokine gradients; however, it’s important to recognize that neutrophils may alter their phenotypes upon exposure to distinct tissue environments (Amulic et al., 2012; Deniset and Kubes, 2018). Circulating neutrophils begin injury resolution in the heart by extravasating the activated endothelium, releasing their noxious granular contents, employing their phagocytic capacity, and forming extracellular chromatin traps rich in inflammatory enzymes (Amulic et al., 2012; Horckmans et al., 2017; Martinod et al., 2017). The content within neutrophil granules can vary, as they are shaped by the transcriptional program present during formation; however, they typically encompass agents like myeloperoxidase, cathepsins, and neutrophil gelatinase-associated lipocalin (NGAL) which help to repress injury through oxidative and proteolytic actions (Amulic et al., 2012). Release of neutrophil granules can occur very rapidly after activation, but neutrophil extracellular traps (NETs) are more insidious. NETs result from slow cell death (NETosis) or non-lytic secretions (Papayannopoulos, 2018). Essentially, released granular proteases like neutrophil elastase (NE) degrade actin polymers, and disrupt chromatin structure; as neutrophil membranes rupture, these intracellular contents expand to the extracellular space (Metzler et al., 2014; Papayannopoulos, 2018). Altogether, these actions make neutrophils very inflammatory, so consequently, as a benefit to the host, they are very short lived cells (Amulic et al., 2012).

Neutrophils have gained traction in the field of cardiac fibrosis given their early prevalence in injuries like MI and myocarditis (Horckmans et al., 2017; Weckbach et al., 2019). Following MI, neutrophils invade the heart in response to necrosing tissue, and they accumulate in the infarct border zone (Askari et al., 2003; Prabhu and Frangogiannis, 2016). They release inflammatory mediators and proteolytic enzymes in order to assist in clearing dead cells and matrix debris (Prabhu and Frangogiannis, 2016). Neutrophils have mostly been regarded as highly inflammatory cells; earlier studies even suggest that their persistence in sterile inflammation may cause further damage to viable cardiomyocytes (Entman et al., 1992; Prabhu and Frangogiannis, 2016). Therefore, recent studies have sought to understand how neutrophil depletion may impact post-MI healing (Horckmans et al., 2017). Interestingly, 1 week following MI, neutralization of neutrophils has been shown to result in worsened cardiac function that is accompanied by enhanced fibroblast activation and excessive collagen deposition (Horckmans et al., 2017). These data suggest that the neutrophil function and secretome may actually be important for mitigating the onset of fibrotic remodeling in a time- and context-dependent manner post-MI. For instance, neutrophils have been shown to persist in post-MI injury due to constant cytokine and DAMP production; these later stage neutrophils function to promote resolution of inflammation (Ma et al., 2016). They express archetypal anti-inflammatory genes and release distinct lipid mediators, which are critical in dampening further pro-inflammatory responses (Serhan et al., 1995; Ma et al., 2016; Prabhu and Frangogiannis, 2016). Through gene expression analysis, further studies have provided evidence to support that neutrophils exist along a phenotypic continuum post-MI; they express pro-inflammatory genes early after injury, but participate in ECM reorganization during post-MI repair (Ma et al., 2016; Daseke et al., 2019). Interestingly, the deletion of an enzyme that catalyzes inflammatory metabolites (12/15 lipoxygenase) promotes anti-inflammatory neutrophil phenotypes post-MI, which also correlates with decreased fibroblast activation and collagen deposition by 5 days post-MI injury (Kain et al., 2018).

Additional studies have characterized the neutrophil-dependent regulation of fibrosis focusing on other pathological states. For instance, neutrophils have been examined for their contribution to fibrosis and ultimately, atrial fibrillation (Friedrichs et al., 2014). After 2 weeks of angiotensin II infusion and deletion of macrophage integrin Mac-1, attenuating the neutrophil infiltration and accumulation within the atrium leads to decreased atrial fibrosis and fibrillation episodes (Friedrichs et al., 2014). Recent studies have also focused on how neutrophil processes like NETosis may impact ventricular fibrotic remodeling after injury. Sustained inflammation, even after pathogen clearance makes myocarditis a major risk factor for developing inflammatory dilated cardiomyopathy (DCM) (Weckbach et al., 2019). NETs have been detected in human cases of myocarditis, which can be replicated in mice using experimental models of myocarditis (Weckbach et al., 2019). Following experimental myocarditis, an increase in NETs corresponds to an increase in collagen deposition, which is alleviated upon neutralizing a cytokine essential to neutrophil recruitment and NET formation (Weckbach et al., 2019). The correlation between NETs and fibrosis is further supported in the context of ageing. Ageing is known to trigger the expansion of neutrophils and NETs; disrupting NET formation in aged mice has been shown to improve cardiac function and decrease ageing related interstitial fibrosis (Martinod et al., 2017). Taken together, neutrophils may serve to regulate fibrosis; however, their actions are clearly context-dependent.

### Monocytes and Macrophages

Since their early discovery, we have come to appreciate the complexity of monocyte and macrophage biology. These leukocytes primarily function as key defenders of the innate immune system by sensing for pathogens, digesting debris, and releasing inflammatory mediators (Hulsmans et al., 2016). However, years of intense scrutiny has revealed numerous other roles for monocytes and macrophages in organ homeostasis and injury, even in the heart (Lavine et al., 2018; Skelly et al., 2018; Williams et al., 2018). Macrophages that are innate to the heart stem mainly from embryonic precursors, with some input from circulating monocytes (Ma et al., 2018; Williams et al., 2018; Dick et al., 2019). These resident cardiac macrophages exhibit distinct gene expression profiles, and have the capacity for self-renewal through adulthood (Skelly et al., 2018; Williams et al., 2018). In the case of injury, circulating monocytes infiltrate and differentiate in the heart; these monocyte/macrophage populations have long been evaluated for their contribution in the fibrotic response. Owing to their remarkable plasticity and heterogeneity, monocytes and macrophages have been assessed for their ability to impart both pro- or anti-fibrotic effects in the myocardium (Wynn and Barron, 2010). For instance, studies have sought to characterize subsets of monocytes/macrophages which may act as additional cells capable of myofibroblast transdifferentiation. Further, numerous studies have also questioned monocytes/macrophages ability to initiate fibroblast activation or aid in resolution of fibrosis through debris clearance mechanisms (Kong et al., 2014). Below, we consider findings of these studies, which suggest subset dependent roles for macrophages and monocytes in cardiac fibrosis.

Until recently, the origin of cardiac myofibroblasts was not entirely clear; however, their induction in response to injury was widely documented (van Amerongen et al., 2008). Earlier findings revealed potential roles for leukocytes in heart regeneration post-MI, and posited leukocytes as cell sources of myofibroblast populations in other organs (Orlic et al., 2001; Forbes et al., 2004). This led cardiac researchers to hypothesize the same phenomena in the context of ischemic injury. To test the leukocyte contribution to myofibroblast transdifferentiation, studies took advantage of bone marrow transplantation with fluorescent-labeled cells (Mollmann et al., 2006; van Amerongen et al., 2008). Following ischemic injury, researchers were able to identify GFP and F4/80 (monocytes/macrophages marker) double positive cells in the infarct, which also co-localized with αSMA (Mollmann et al., 2006; van Amerongen et al., 2008). Importantly, the lack of CD31 co-localization suggested these cells were independent of the vasculature (Mollmann et al., 2006). Further analyses of these bone marrow derived myofibroblasts even suggested that they partake in collagen production (van Amerongen et al., 2008). Indeed, a recent study using myeloid-specific cre reporter mice (LysM^*Cre/*+^; ROSA26-eYFP) mice has suggested that macrophages can indeed transition toward fibroblast-like cells in the heart following MI with respect to their expression profile of fibroblast markers over time (Haider et al., 2019). However, additional genetic lineage tracing studies using similar reporter mice (LysM^*Cre/*+^; Rosa26-eGFP) showed negligible contribution of monocytes and macrophages to myofibroblasts post-MI, which suggest that myofibroblasts derive largely from proliferating and transdifferentiating resident cardiac fibroblasts (Kanisicak et al., 2016).

In the injured heart, infiltrating monocytes differentiate into macrophages near spaces rich with collagen producing myofibroblasts; these macrophages can then influence the surrounding cells and ECM through numerous mechanisms (Pappritz et al., 2018; O’Rourke et al., 2019). For instance, activated macrophages within the injured myocardium have been shown to contribute to fibroblast activation, proliferation and myofibroblast transformation via secretion of pro-fibrotic factors, including transforming growth factor-beta (TGFβ) and galectin-3 (Gal3) (de Boer et al., 2009; Hundae and McCullough, 2014). Additionally, macrophage phagocytosis can often be a trigger in regulating fibrosis (Kim et al., 2017), where macrophage ingestion of dead cells and debris leads to the release of pro-fibrotic mediators including TGFβ (Nacu et al., 2008). However, macrophage phagocytic activity may also contribute to resolution of fibrosis; their ability to take up myofibroblasts and matrix debris may function to reduce the level of fibrotic stimuli (Wynn and Barron, 2010; Kong et al., 2014). Macrophages also function to secrete inflammatory mediators, which regulate fibroblast activation or ECM remodeling. In detail, activated macrophages can secrete cytokines and factors like interleukin (IL)-1β, tumor necrosis factor α (TNFα), fibroblast growth factor (FGF) and platelet derived growth factor (PDGF) which directly activate fibroblast transdifferentiation, or contribute to increased production of matrix metalloproteinases (MMPs) (Kaikita et al., 2004; Kong et al., 2014; O’Rourke et al., 2019). Increased production of MMPs leads to excessive matrix remodeling which disrupts fibrotic remodeling (Yan et al., 2006; Halade et al., 2013; Aghajanian et al., 2019). Additional associative studies, particularly focusing on epicardial adipose tissue (EAT) and its role in atrial fibrillation have hinted macrophages involvement in fibrosis (Abe et al., 2018). Specifically, an increase in macrophage density in EAT is positively associated with severe EAT fibrosis, which correlates with enhanced atrial fibrosis (Abe et al., 2018). Importantly, patients with atrial fibrillation exhibited greater severity of fibrosis (Abe et al., 2018). Altogether, these findings provide evidence for the opposing fibrogenic actions of macrophages and its secretome, which suggests the need for careful context dependent analysis when targeting macrophage function in the fibrotic response.

In recent years, much attention has been focused upon tissue resident monocyte/macrophage populations, which function uniquely in the post-injury repair process. For instance, studies have identified distinct peritoneal Gata6 macrophage subsets that are critical for the resolution of inflammation (Rosas et al., 2014). Gata6 is thought to be essential to these macrophages phenotype, and is key to their ability to self-renew and persist through homeostasis and inflammation (Rosas et al., 2014). Notably, Gata6 macrophages have been identified in the pericardial cavity of both mice and humans, and they have been shown to regulate cardiac fibrosis following cardiac injury (Deniset et al., 2019). Four weeks following MI, myeloid-specific deletion of Gata6 resulted in enhanced fibrosis especially in the remote myocardium (Deniset et al., 2019). These findings suggest the benefits of pericardial macrophages, namely in preventing adverse fibrotic remodeling in viable tissue. Further studies have sought to characterize how resident macrophage subsets influence fibrosis in cardiac injury. For instance, depletion of CCR2^+^ resident macrophages prior to a model of ischemia/reperfusion resulted in decreased fibrosis (Bajpai et al., 2018), while depletion of CX3CR1^+^ resident macrophages led to worsening fibrosis, remodeling and functional outcomes (Dick et al., 2019). Considering these findings, it is clear that resident macrophages exert important effects on post-injury cardiac remodeling outcomes, potentially through the regulation of fibrotic responses.

### Mast Cells

Mast cells (MC) are most recognized for their function in regulating allergic responses; however, years of thorough investigation has revealed their much more considerable and widespread involvement in the immune system (Rao and Brown, 2008; da Silva et al., 2014). These secretory leukocytes are characterized by their abundant and diverse granules, which contain a combination of preformed cytokines, growth factors, and proteases (da Silva et al., 2014). In the absence of injury, MCs reside in low density in tissues like the heart; they originate from bone marrow derived precursors that migrate and subsequently mature into phenotypes dependent on their microenvironments (Levick and Widiapradja, 2018). Injury triggers additional MC precursors to infiltrate the myocardium (Ngkelo et al., 2016). For instance, numerous reports have shown increased mast cell counts in DCM patient hearts and animal models of cardiac injury like hypertension and MI (Sperr et al., 1994; Engels et al., 1995; Patella et al., 1997; Shiota et al., 2003; Janicki et al., 2015). This increase in mast cells following injury has led many researchers to question their involvement in cardiac fibrotic remodeling.

To date, a plethora of compelling results suggest roles for MCs in cardiac fibrosis (Legere et al., 2019). For one, in injury, the presence of DAMPs can trigger MC degranulation, which results in the release of inflammatory mediators like tryptase, chymase, IL-1β, and TNFα (Legere et al., 2019). Studies have shown that both tryptase and chymase activate TGFβ, a potent fibrogenic mediator known to promote myofibroblast differentiation and collagen production (Lindstedt et al., 2001; Tatler et al., 2008; Legere et al., 2019). Similarly, inflammatory cytokines including TNFα have been implicated in the development of cardiac fibrosis during hypertension (Mayr et al., 2016). MC are also direct sources of fibrogenic mediators. For one, MC have been implicated in atrial fibrosis and fibrillation through enhanced production of the pro-proliferative and fibrotic factor PDGF-A (Liao et al., 2010). MC granules have also been shown to contain fibroblast growth factor-2 (FGF2), which has been shown to positively regulate fibroblast proliferation and collagen production (Hamada et al., 2000; Virag et al., 2007; Wernersson and Pejler, 2014). As degranulation products have been shown to induce fibrosis, recent studies have assessed the impact of inhibiting this inflammatory release. Interestingly, treatment with MC stabilizers has been shown to reduce collagen deposition, and collagen gene expression (Wang et al., 2016). Further, recent studies focusing on pressure overload induced injury have also identified an association between increasing mast cell volume and fibrosis (Luitel et al., 2017). These results imply that MCs function to promote adverse fibrotic remodeling; however, arriving at that conclusion may not be accurately reflective. Mice lacking mast cells exhibit only marginally decreased fibrosis 2 weeks after MI (Ngkelo et al., 2016). These finding suggest insignificant roles for mast cells in fibrosis, however, interestingly, there is also evidence, which proposes mast cells as anti-fibrotic mediators. Following induction of cardiac dysfunction, mast cell deficient mice show increased perivascular fibrosis (Joseph et al., 2005). Further, MCs have been shown to produce anti-inflammatory agents including IL-10 which act against fibrosis (Lin and Befus, 1997). Specifically, IL-10 has been repeatedly noted for its ability to oppose fibrosis through reducing collagen gene expression and fibrotic scarring after injury (Krishnamurthy et al., 2009; Verma et al., 2017). In sum, the current understanding of the role of mast cells in fibrosis is not entirely clear, which necessitates further studies to address the inconsistencies seen throughout the literature.

### Eosinophils

Eosinophils are a subset of circulating leukocytes that contribute to the clearance of injury primarily through their hydrolase rich granules (Seguela et al., 2015). Recent focus has been placed on this leukocyte subset as patients exhibiting high levels of eosinophils (eosinophilia) can be at risk for cardiac complications (Diny et al., 2017; Prows et al., 2019). This clinical phenomenon is supported by recent animal studies that characterize cardiac remodeling in eosinophilia, and see a large response featuring replacement fibrosis (Prows et al., 2019). Specifically, mice that develop eosinophilia experience a large influx of eosinophils to the heart, along with enhanced chemokines and cytokines which may function to promote fibrosis (Prows et al., 2019). Beyond these findings, additional studies have shown a correlation between enhanced eosinophil accumulation and cardiac fibrosis. Following experimental myocarditis, the depletion of natural killer (NK) cells leads to an accumulation of eosinophils with a concomitant rise in fibrosis (Ong et al., 2015). These findings suggest that eosinophils contribute to cardiac fibrosis through cytokine secretion and cellular cross talk; however, more evidence is required in order to understand the exact contribution of eosinophils to cardiac fibrosis. Some studies have begun to address these needs through eosinophil depletion studies. In a model of myocarditis, a lack of eosinophils prevents against the development of inflammatory DCM (DCMi) (Diny et al., 2017). While there are no significant changes in total ventricle fibrosis, eosinophil depletion post-myocarditis leads to decreased expression of MMP2 and tissue inhibitor of metalloproteinases 2 (TIMP2) (Diny et al., 2017). These reports highlight the need for definitive assessment of the mechanisms by which eosinophils may trigger cardiac fibrosis.

### Dendritic Cells

Dendritic cells (DC) are key immune regulators that function to initiate adaptive responses through antigen processing and presentation (Durai and Murphy, 2016). They can be characterized as being classical or non-classical, both of which exist in numerous subsets (Mildner and Jung, 2014). DCs are highly abundant in barrier tissues like the skin, however, many reports have shown their presence in heart tissue (Van der Borght and Lambrecht, 2018). Specifically, recent studies have suggested at least two types of classical or conventional DCs (cDC) in the healthy heart (Van der Borght et al., 2017). These cDCs have been shown to mediate tolerance to self-antigens (Van der Borght et al., 2017). DCs become of even greater relevance as the heart experiences injury (Clemente-Casares et al., 2017; Van der Borght et al., 2017; Wang et al., 2017). Their specific roles in pathology, especially in relation to cardiac fibrosis, is distinct; we consider them below.

A few studies have highlighted an influx of CD11c^+^ cDCs to the heart following ischemic injury that increase starting 1 day post-MI that peak around 5–7 days post-MI (Anzai et al., 2012; Van der Borght et al., 2017). This early rise in DCs has led researchers to hypothesize additional roles for DCs in repair and remodeling post-injury. Accordingly, models of CD11c^+^ depletion have been applied, and following MI, a lack of bone marrow derived CD11c^+^ cells results in decreased survival rates and significantly enhanced fibrosis (Anzai et al., 2012). Although this model relies on the non-specific loss of all CD11c^+^ cells, these findings provided evidence for DCs as being somewhat protective after infarction. Interestingly, this protective DC theory can be further supported by studies analyzing DC densities in humans post-MI or in DCM (Pistulli et al., 2013; Nagai et al., 2014). For instance, patients with DCM have decreased DCs compared to controls, and experience enhanced fibrosis and worsened cardiac function (Pistulli et al., 2013). There are also additional studies, focused on myocarditis, which suggest protective DC roles following injury (Clemente-Casares et al., 2017). A specific deletion of cardiac CD103^+^ DCs results in significantly enhanced fibrosis following viral myocarditis, which suggests the role of DCs in blunting the transition to HF following injury (Clemente-Casares et al., 2017).

Beyond these findings, however, it is also important to note the wealth of literature that hints at an opposing role for DCs following injury. With CD11c^+^ cell depletion, 24 weeks after TAC, mice exhibit reduced LV fibrosis and decreased expression of fibroblast activating genes (Wang et al., 2017). These studies are met with similar critiques regarding lack of DC specificity, however, studies using a more specific model of cDC ablation also show decreased LV fibrosis 3 weeks post-MI (Lee et al., 2018). These findings are further supported by earlier studies which show cardiac and splenic DC activation and expansion 8 weeks post-MI (Ismahil et al., 2014). Interestingly, transfer of splenocytes from these HF mice into naïve mice results in the development of HF accompanied with significant fibrosis (Ismahil et al., 2014). Taken together, these studies provide evidence for DCs in post-injury remodeling, however, there is limited insight into how DCs may influence fibrosis. Overall, consideration of findings from each of these studies suggests a continued need for further DC characterization in the heart, focusing on how unique and specific populations of DCs may act to govern the fibrotic response.

### B and T – Lymphocytes

An abundance of literature suggests an involvement of both B and T– Lymphocytes (B and T cells) in cardiac fibrosis. These adaptive immune cells are found in small numbers in the steady state heart, but are known to rise post-injury (Zouggari et al., 2013; Wang et al., 2019). The functional relevance of these lymphocytes in the steady state heart is still becoming understood, however, following injury, several reports have documented their ability to influence long-term remodeling outcomes (Yu et al., 2013; Zouggari et al., 2013; Horckmans et al., 2018). For instance, B cells have been shown to be associated with enhanced fibrosis; their depletion results in reduced collagen deposition following TAC, MI, and the development of non-ischemic cardiomyopathy (Cordero-Reyes et al., 2016; Horckmans et al., 2018; Ma et al., 2019). B cells are thought to promote fibrosis through inflammatory cytokines like IL-1β, IL6, and TNFα, all of which have been linked to increased fibrosis (Cordero-Reyes et al., 2016). Interestingly, these findings are supported by other studies that identify increased B cell densities and cardiac fibrosis in DCM patients when compared to healthy controls (Yu et al., 2013). B cells isolated from these DCM patients secrete larger quantities of pro-fibrotic factors including TNFα; these findings provide additional evidence to support the pro-fibrotic actions of B cells in injury (Yu et al., 2013). In moving forward, much remains to be understood in the field of cardiac B cell biology, including the explicit role that B cells play in the uninjured heart, as well as the mechanisms by which they elicit pathological fibrotic remodeling.

Similar to B cells, T cells have been shown to infiltrate the injured myocardium where they greatly influence remodeling outcomes (Blanton et al., 2019). T cells come in many subsets, some of which have divergent functions in cardiac fibrosis. For one, CD4^+^ T cells accumulate in the heart post-MI and -TAC, and are associated with enhanced cardiac fibrosis (Laroumanie et al., 2014; Nevers et al., 2015, 2017; Bansal et al., 2017, 2019; Borg et al., 2017; Quast et al., 2017). Studies have shown increased Th1 polarization following injury, leading to the production of pro-inflammatory and -fibrotic cytokines like interferon γ (IFNγ) (Laroumanie et al., 2014; Nevers et al., 2017). As these subsets of T cells are implicated in worsening fibrosis, many reports have sought to characterize their direct involvement in the remodeling response. The depletion or dampening of T cell infiltration post-injury has been shown to result in a significant reduction of fibrosis and fibrosis associated gene expression (Laroumanie et al., 2014; Nevers et al., 2015; Salvador et al., 2016). Recent studies have also shown a direct increase in cardiac fibrosis following adoptive transfer of HF splenic CD4^+^ T cells into naïve mice (Bansal et al., 2017). Mechanistically, a few targets have been investigated in their role for inducing pro-fibrotic T cell actions. Namely, studies have determined an involvement of CD73 and adenosine generation, as T cell-specific CD73 knockout mice show enhanced fibrosis (Borg et al., 2017; Quast et al., 2017). These studies suggest that adenosine functions in an autocrine manner to reduce the production of pro- inflammatory and -fibrotic cytokines (Borg et al., 2017). Further, signaling through the mineralocorticoid receptor (MR) has been shown to enhance pro-fibrotic T cell actions, as the loss of T cell MR has been shown to decrease T cell activation and fibrotic cytokine production (Li et al., 2017). Interestingly, all of these findings seem to be associated with CD4^+^ T cells, as similar assessments have revealed no significant role for CD8^+^ T cells in cardiac fibrosis (Groschel et al., 2018).

Studies have also examined the role of T regulatory (Treg) subsets in fibrosis, and have mainly shown their role in protecting against fibrosis (Kvakan et al., 2009; Kanellakis et al., 2011; Tang et al., 2012). Early studies have shown decreased collagen content and lower expression of pro-fibrotic mediators following Treg introduction into hypertensive injury models (Kvakan et al., 2009; Kanellakis et al., 2011). Additionally, the introduction of Tregs post-MI has also been shown to reduce border zone fibrosis, further suggesting a protective mechanism through which Tregs influence remodeling (Tang et al., 2012). However, recent studies have shown alternative Treg functions post-injury, wherein an increase in a pro-inflammatory CD4^+^ Treg subset has been shown to lead to the production of fibrotic cytokines like TNFα (Bansal et al., 2019). Additional studies directed toward the characterization of the impact of specific T cell subsets on cardiac fibrosis at distinct timepoints after injury will be helpful to illuminate potential targets against pathological cardiac fibrosis.

## Concluding Remarks

Fibrotic remodeling is a normal physiological response to injury in the heart, however, when it becomes excessive, fibrosis leaves the heart at a great detriment in that it leads to tissue stiffness and dysfunction (Hinderer and Schenke-Layland, 2019). As such, the prevention or reversal of pathological fibrotic remodeling has been a research target for numerous years and has led to a wealth of studies aimed in uncovering the mechanistic basis for cardiac fibrosis. Recently, studies using isolated myofibroblasts from HF patients have suggested the possibility of reversing fibrosis by inhibition of TGFβ receptor signaling (Frangogiannis, 2019b; Nagaraju et al., 2019). These findings provide support to reason the idea of myofibroblasts returning to quiescent fibroblast states (Nagaraju et al., 2019). However, translating these findings *in vivo* will require consideration of numerous other factors which influence cardiac fibrosis. For one, several leukocyte subsets are implicated in the development of fibrosis. Currently, their explicit contribution to cardiac fibrosis, which appears context-dependent, is still being unraveled, as is the practicality of targeting specific subtypes at particular phases of post-injury remodeling of HF development. For instance, the presence of dendritic cells may be beneficial toward stable scar formation in MI patients, however, their exact role in the regulation of fibrotic remodeling and how to support their activity will need to be understood to effectively translate this knowledge toward therapeutic application (Nagai et al., 2014). Further, patients with non-ischemic HF have been shown to exhibit higher numbers of activated CD3^+^ T cells; given the number of animal studies showcasing their association with fibrosis, antagonizing these lymphocytes could serve as a feasible therapeutic avenue against pathological fibrosis during HF (Nevers et al., 2015). To conclude, a growing body of evidence suggests multifaceted roles for leukocytes in cardiac injury: both innate and adaptive leukocytes influence pathological fibrotic remodeling, presenting an exciting trajectory for the development of novel therapeutic strategies that modulate their recruitment, secretions and phenotypes to prevent the progression of both ischemic and non-ischemic forms of HF.

## Author Contributions

AO and DT wrote the manuscript.

## Conflict of Interest

The authors declare that the research was conducted in the absence of any commercial or financial relationships that could be construed as a potential conflict of interest.
